# QALY-time: experts’ view on the use of the quality-adjusted LIFE year in COST-effectiveness analysis in palliative care

**DOI:** 10.1186/s12913-020-05521-x

**Published:** 2020-07-16

**Authors:** Anne B. Wichmann, Lia C. M. J. Goltstein, Ndidi J. Obihara, Madeleine R. Berendsen, M. Van Houdenhoven, R. Sean Morrison, Bridget M. Johnston, Y. Engels, on behalf of the Radboud Honours Academy Think Tank, Madeleine Berendsen, Lia Goltstein, Elze Knol, Melvin Kool, Wytse Nienhuis, Luc Nies, Ndidi Obihara, Jelte Pieksma, Jordy Rovers

**Affiliations:** 1 Radboud university medical centre, Department of Anaesthesiology, Pain and Palliative Medicine, Nijmegen, The Netherlands; 2 Radboud University, Honours Academy, Nijmegen, The Netherlands; 3 Sint Maartenskliniek, Ubbergen, The Netherlands; 4 Icahn School of Medicine at Mount Sinai, New York, USA; 5 Trinity College Dublin, Centre for Health Policy and Management, Dublin, Ireland

**Keywords:** Quality-Adjusted life years, Cost-benefit analysis, Palliative medicine, Focus groups (MeSH)

## Abstract

**Background:**

The Quality-Adjusted Life Year (QALY) is internationally recognized as standard metric of health outcomes in cost-effectiveness analyses (CEAs) in healthcare. The ongoing debate concerning the appropriateness of its use for decision-making in palliative care has been recently mapped in a review. The aim was to report on and draw conclusions from two expert meetings that reflected on earlier mapped issues in order to reach consensus, and to advise on the QALY’s future use in palliative care.

**Methods:**

A nominal group approach was used. In order to facilitate group decision making, three statements regarding the use of the QALY in palliative care were discussed in a structured way. Two groups of international policymakers, healthcare professionals and researchers participated. Data were analysed qualitatively using inductive coding.

**Results:**

1) Most experts agreed that the recommended measurement tool for the QALYs ‘Q’ component, the EuroQol-5D (EQ-5D), is inappropriate for palliative care. A more sensitive tool, which might be based on the capabilities approach, could be used or developed. 2) Valuation of time should be incorporated in the ‘Q’ part, leaving the linear clock time in the ‘LY’ component. 3) Most experts agreed that the QALY, in its current shape, is not suitable for palliative care.

**Conclusions:**

1) Although the EQ-5D does not suffice, a generic tool is needed for the QALY. As long as no suitable alternative is available, other tools can be used besides or serve as basis for the EQ-5D because of issues in conceptual overlap. 2) Future research should further investigate the valuation of time issue, and how best to integrate it in the ‘Q’ component. 3) A generic outcome measure of effectiveness is essential to justly allocate healthcare resources. However, experts emphasized, the QALY is and should be *one* of multiple criteria for choices in the healthcare insurance package.

## Background

Healthcare costs are rising due to expanding treatment options, the aging of the baby boomers, and increased longevity [1–3]. This increasingly raises questions as to which medical interventions should be financed and which not [4–6]. In order to answer this question and to allocate resources efficiently and fairly, evaluation of the relative costs and effectiveness of interventions in comparison to alternatives is needed [5]. Cost-effectiveness is not the only basis for allocation decisions; in the Netherlands for example, it is one of four criteria in the insurance coverage process [7]. Nevertheless, it is becoming increasingly important. The standard outcome metric in cost-effectiveness analysis to inform decisions on resource allocation in health care is the Quality-Adjusted Life Year (QALY). The basic idea underlying the QALY is simple: one year of life lived (‘LY’) in perfect quality (‘Q’) is worth one QALY. The more added life years and quality of life (QoL) during this period, compared to an interventions’ best alternative, the higher the number of QALYs added by a certain intervention. Subsequently, societies can determine the willingness to pay for a QALY gained. In this way, the relative costs and outcomes of interventions can be compared.

However, there is an ongoing debate regarding the appropriateness of the use of the QALY in palliative care **[**8**–**10**].** Among others because of the complexity of needs of palliative patients, which can be diverse and vary from symptom relief to information needs and autonomy to make decisions, to psychosocial support for coping with their disease, or spiritual and existential questions **[**11**].**.

The QALY debate in palliative care was recently mapped in an integrative review **[**12**].** In three themes, this review described which problems are encountered when using the QALY in the palliative care context: restrictions in life years gained; conceptualization of QoL and its measurement including suggestions to adapt this, and; issues around valuation and additivity of time, referring to changing valuation of time. In this review, it among others was concluded that the QALY might be more valuable for palliative care when QoL questionnaires taking into account issues important to palliative patients (such as patient dignity, spiritual and psychosocial wellbeing and closure) are used, and that the possibility of integrating valuation of time in a non-linear way in the QALY should be explored.

Since the field is moving forward, and in order to take the next step in this debate, in this study we brought together diverse stakeholders in order to have a conceptual discussion – overarching economic arguments – regarding the use of the QALY in palliative care; where are we going? The aim of this article was to report on, and draw conclusions from these international meetings in which experts reflected upon the abovementioned issues in order to reach consensus on the identified QALY issues, [12] and to advise on the QALY’s future use in practice and policy.

## Methods

### Design

A nominal group technique (NGT) was applied. The NGT is a structured method for identifying problems and generating possible solutions for (healthcare) problems using four key stages: silent generation, round robin, clarification and ranking [13]. In reporting on our study, the COREQ- guideline was used [14]. As this study was not subject to medical research involving human subjects, ethical approval was not required.

### Participants

Since the discussion about the use of the QALY has policy, ethical, economic and medical elements, experts with different backgrounds were invited to participate (Table 1). This multidisciplinary approach facilitated a way to collaborate on mutual concerns. Participants were approached by email, telephone or in person. All researchers were present during the NGT. For background information of the researchers, see Table 2. Except for work-related connections, no relationships were established prior to study commencement. Participants were informed about the field of interest of the researchers, and about the particular project.

**Table 1:** Number and characteristics experts

Primary occupation	Session 1	Session 2	Total
Policy maker		0	**6**
*Governmental advisor (economist)*	1		
*Health care director*	5		
Medical professional			**5**
*Medical specialist*	2	1	
*GP*	2		
Researcher (social health or health economics)	1	5	**6**
**Total**	**11**	**6**	**17**

**Table 2:** Background researchers

Researcher	Background
Anne B. Wichmann	Health scientist and ethicist. PhD candidate in economics of palliative care on an EU funded project (PACE)
Yvonne Engels	Associate professor in timely palliative care
Lia C.M.J. Goltstein	Trainee doctor and PhD candidate Internal Medicine
Ndidi J. Obihara	Trainee doctor Surgical Ward
Madeleine R. Berendsen	Phd candidate Medical Biology

### Procedure of the nominal groups

Proposition regarding the main issues of the QALY discussion originating from the earlier mentioned review, [12] formed the rounds of the NGT. They were:

I) The ‘Q’ component: *“The EQ-5D is suitable for palliative care”*
II) The ‘LY’ component: *“The QALY should take non-linearity of time into account”*
III) The QALY in total: *“The QALY is appropriate for palliative care”*

Each round of the NGT included eliciting ideas and arguments around these propositions from the key stakeholders, to subsequently collectively reduce those arguments. Given the complexity and political sensitivity of the QALY problem, a modified version of the NGT was used. Individual expert arguments were not ranked, but collectively reduced to key arguments.

### The international nominal group

Experts from the Netherlands, the U.S., the U.K., Ireland and Belgium participated in the NGT divided into two sessions. The first session was held in March 2017 in the Netherlands and took about 3 hours. The meeting consisted of three rounds and was chaired by experienced moderators Y.E. and A.B.W. In the first two rounds, experts silently reflected and wrote down their individual ideas in response to the proposition. Then, each expert shared his or her opinion and argumentation with the group members (round robin). Arguments were collected on a flip chart by one of the moderators. After voicing all answers and argumentations, all points were clarified if needed, discussed and merged. These key arguments were discussed again. In the third round, main arguments of the first two rounds were written on the flip chart, to include them in the final discussion. The second session was organized at the European Association of Palliative Care (EAPC) conference in Madrid in May 2017, was chaired by Y.E. and A.B.W. and lasted 1,5 h. This meeting started with a summary of the findings of round I and II of the first NGT session. Then, these findings from the first session were refined and complemented by repeating round III. Since the discussion in round III also encompasses issues regarding propositions of round I and II, these issues were also discussed.

### Analysis

Two researchers kept detailed minutes of the meetings. Names of experts were anonymised. Although predefined propositions were used during the analysis phase, all opinions and arguments were constantly openly compared. Participants were asked for explanatory notes if needed. Inductive conventional content analysis using coding, ordering, and clustering was conducted in ATLAS.ti (version 7) to identify patterns that could be translated into themes [15]. Although some analysis was done in between the two meetings, the final analysis took place after the second meeting. Coding was done by A.B.W. and L.C.M.J.G. independently and in multiple rounds. If necessary, codes were discussed amongst A.B.W. and L.C.M.J.G. until consensus was reached.

## Findings

Seventeen participants took part in the NGT (Table 1). Some of the experts had dual roles, e.g. as clinician and policy advisor. Experts invited who declined to take part, mainly did so because of time issues or because they could not attend the conference.

### ROUND I. palliative care and the EQ-5D

#### Generic tool needed

Experts mentioned that an advantage of the EQ-5D is that it is a generic and simple to use tool. Moreover, *“reasoning from resource allocation management, this metric is certainly suitable because one metric is needed. Otherwise there’s no end to it. Where does that leave us?*” (P5). At the same time, experts argued, palliative care is significantly different from other fields of healthcare. Values important in palliative care, such as psychosocial and spiritual, are missing in standard ways of measuring health-related (HR) QoL, and “*by lumping everything together, you’re not measuring anything”* (P11). More than that, it was added in the international meeting that *“in the UK, the EQ-5D is advocated, but they acknowledge that there are limitations to it. They justify using it by not using it blindly”* (E5) In other words: assessing whether the EQ-5D is appropriate is always needed.

#### From specific to generic: mapping

Next, it was mentioned that other, specific palliative care related instruments could be used in practice, and subsequently translated into EQ-5D scores or ‘utilities’. In health economics, this method is known as mapping [16]. Mapping techniques are conducted to link outcomes from different measures, by developing algorithms to translate disease-specific measurement outcomes into EQ-5D utility values. In other words: context specific instruments are used as ‘under layer’ for the EQ-5D. In that way, QoL is measured as a ‘pyramid’ in which the EQ-5D is the top. This makes it applicable on individual as well as on policy level. Experts expressed this option of mapping is very welcome since *“we have to make a translation from individuals to policy. ( …*) *then you need an instrument in which, if the patient looks up* [in the pyramid, added by authors] *thinks ‘well, I can see myself in this’”* (P1).

### Subjective valuation

Another point raised was the lacking subjective valuation in the EQ-5D. That is: how important are the EQ-5D’s domains to patients? *“I* [a general practitioner] *miss the person’s context and his or her goals. For me, that are essential questions which I discuss with patients ( …*) *that’s why I would advocate to explicitly address the meaning people give to the domains in these questionnaires. Otherwise, the patients’ interpretation is totally unclear”* (P1). Another expert described it as follows: *“if we are limping with one leg and are happy, then the limping doesn’t matter!”* (P11)

The QALY’s ‘Q’ could be defined in a different concept of disease and health, it was argued, such as the capabilities approach [17]. Here, measuring QoL is conceptually linked to Sen’s theory, which defines wellbeing in terms of an individual’s ability to *be* and *do* the things that are important in his or her life [18]. Others though, saw some drawbacks, since measuring capabilities is not the same as measuring health. “*However, when considering Huber’s new definition of positive health* [health as the ability to adapt and self-manage, in the face of social, physical and emotional challenges] *it might be appropriate”* (P1).

### ROUND II. The QALY and linearity in valuation of time issues

#### Time linearity

Initially, a discussion about linearity of valuation of time took place. Kahneman’s Peak-End Rule was discussed [19]. His theory showed that valuation of time (*Kairos*) is not linear, but that valuation of experiences are mostly influenced by most intense points (positive or negative) and their end. Translating this to the palliative care context: when approaching death, a distortion of how time is experienced and valued takes place. One expert mentioned that “*there is no linearity* [in valuation of time]*. It is not only not there in palliative care, it is absent in everything. So, is it specifically a palliative care problem?”* (E3).

#### Chronos and Kairos

However, experts noted the issue about linearity of valuation of time is not as relevant an issue for the QALY if a distinction is made between *Chronos* (clock time) and *Kairos* (embodied time) [20]. Because *“perception and valuation of time should be integrated in the QALY’s ‘Q’ … Clock time should serve as an absolute basis for how you value time. Valuation belongs in the ‘Q’!”* (P8). In other words: *“Chronos is clock time, Kairos is quality time: the time that’s so important to people”* (P9). Experts noted that further research should be conducted into the issue of linearity of valuation of time, and how to integrate it in the QALYs ‘Q’.

Furthermore, it was emphasized it should be possible to denominate *Kairos* negatively. It was also posited that palliative care only involves *Kairos*, not *Chronos*. However, the majority of experts disagreed with this statement, since this is different for everyone and *“living longer* [Chronos] *may be part of QoL”* (P1). However, the idea of putting different weights on time during the disease trajectory was proposed since *“towards the end of life, the life-years-sum becomes increasingly smaller, and the Q starts to play an increasingly important role … somewhere along the route you lose sense”* (P7).

### ROUND III. Applicability of the QALY in palliative care

#### Generalisability

Some experts, as opposed to earlier arguments with regard to the EQ-5D, argued we should *“not try to put everything in one frame* [the QALY in general]*, but instead compare different frameworks for different groups”* (E1). The QALY has a simplistic approach by merging all sorts of health care in one framework. But should we be even comparing across groups?

#### QALY in policy

It was concluded that society needs a concept like the QALY, since cost-effectiveness is and should be considered when deciding on how to distribute resources over health care. However, it was also noted we might not want to put a hard threshold on the worth of a QALY, but instead use it as guidance. “*In the U.K. a threshold of £20.000/£30.000 is set, but it is constantly disregarded because of political difficulties in drawing a strict line”* (P5). In the Netherlands, an advising committee has ethical discussions based on various figures, *“all grey areas are being discussed in these meetings. And they have to. Because doing that exactly is the rationale of its existence*” (P5). In other words; the QALY is not and should not be used as a technocratic tool, but as part of a broader, very thorough assessment.

Moreover, *“the question about how much one QALY is worth, is still in full swing”* (P5). For example, the Dutch Council for Public Health and Health Care (an independent advisory body of the Ministry of Health, RVZ) advised a threshold of €80,000 per QALY. *“However, this threshold was not adopted by the minister, because of a lack of support in the ministry”* (P3).

## Discussion

### Main findings

By means of systematic international expert discussions, a conceptual discussion regarding the use of the QALY in palliative care was held in this study. Earlier identified issues [12] were reflected upon and discussed with international experts in order to reach consensus on these issues, and to advise on the QALY’s future use in policy and practice. These issues among others concerned the QALY possibly being more valuable for palliative care when QoL questionnaires used take into account issues important to palliative patients (such as patient dignity, spiritual and psychosocial wellbeing and closure), and the possibility of integrating valuation of time in a non-linear way in the QALY.

### Quality of life: the QALY’s ‘Q’ component

Experts largely agreed that the recommended EQ-5D for measuring the QALY’s ‘Q’ component is inappropriate for palliative care. They emphasized that a generic tool is needed though, and as long as no suitable alternative is available, other measurement instruments can be used additionally, so that utilities can still be calculated. And although research indicated that the EQ-5D-5L seems to increase sensitivity and precision, as it measures five instead of three response options (EQ-5D-3L) to the five EQ-5D dimensions [21], this does not solve the problem regarding the domains covered. Because, as a recent study assessing the level of conceptual overlap between the palliative outcome scale (POS) and the EQ-5D showed [22], the EQ-5D is unlikely to provide an appropriate basis for estimating utilities when conducting economic evaluations in palliative care.

To solve the ‘generic tool problem’, an expert proposed to map a specific palliative care instrument onto the EQ. 5D in order to obtain utilities for the QALY, a technique which has been applied in earlier research [16, 23, 24]. When doing this, important domains are measured on micro level, which subsequently can be translated to ‘better informed’ EQ-5D utilities useful on macro (policy) level. However, the above mentioned issue regarding conceptual overlap remains problematic: the EQ-5D is unlikely to provide an appropriate basis for estimating utilities when conducting economic evaluations in palliative care. Therefore, it is unlikely that the palliative care measure will map closely to the EQ-5D. We therefore suggest future research to look into possibilities of mapping for the palliative care context.

Moreover, when defining and measuring wellbeing in terms of an individual’s ability to *be* and *do* the things that are important in his or her life, instead of measuring functioning, the issue of “*if we are limping with one leg and are happy, then limping doesn’t matter*” would be of less concern. And although some argue in favour of a generic approach towards valuing health states [25], relying on societal values in calculating utilities is seen as a limitation by others, as it causes overestimation of disutility. Research indicates that the general, healthy population is not able to validly predict what they will value when the end of their lives are nearing [26, 27]..

### The QALY and linearity in valuation of time issues: Chronos and Kairos

Experts in this study argued that the time issue might not be of considerable concern for the ‘LY’ component since valuation of time belongs in the ‘Q’ component. Linear clock time (*Chronos*) is kept in the ‘LY’ component: a component which according to experts can definitely be important in palliative care. It was argued though, that the preference weight put on both components (‘Q’ and ‘LY’) can shift during the disease trajectory, and that this might be considered in future use of the QALY in palliative care.

The time trade-off (TTO) valuation technique is widely used to determine utility values of health outcomes [28]. When using TTO, time spent in a health state is valued, based on general population time relativity. However, these calculated utilities do not consider different *life phases*, nor the fact that there are circumstances where people put more or less value on time. At the same time, Kahneman in his Peak End Rule described that the way people evaluate past experiences tends to be based on the most intense points (best or worst) and how they end [19]. This issue is currently disregarded in the EQ-5D [19, 29].

The issue of valuation of time has been debated extensively in the literature, and is still ongoing. Some argue the QALY’s use is controversial because of its underlying assumption that each time segment is equally weighted, making them additive [28–30]. However, it is shown that there are circumstances where people put more or less value on time, for example during peaks and the end of an experience [1]. Authors using this Peak End Rule theory, argue that people approaching death encounter a profound distortion in how time is experienced and valued, [2] and that whole experiences are marked by how they end. Moreover, a recent study concluded that time perception of terminal patients with cancer indeed differed from the time perception in earlier phases of life [3]. However, as was shown in a BMJ paper, prognosis is often difficult to estimate [27]. This difficulty of prognostication makes studying valuation of time challenging, which should be rightly acknowledged in future research. Others claim that if time at the end of life is valued extra, an equity weight should be applied to these segments of time to estimate QALYs [31].

### Applicability of the QALY in palliative care

Finally, it was concluded that even though the QALY currently does not suffice for palliative care, an instrument is needed to justly allocate health care resources. The suggestion of using different frameworks for different groups, such as the ‘PALY’ concept for palliative care interventions [30], could be further explored in future research. The debate regarding the use of a strict threshold for one QALY is also found in the literature [31, 32]. We suggest future studies to focus on these issue.

### Strengths and weaknesses

As the discussion about the use of the QALY in palliative care has ethical, economic and medical elements, a strength of this study is its multidisciplinary character. Moreover, the participation of international experts strengthens its eloquence. Furthermore, its systematic NGT approach enabled reaching consensus and practical advises on the QALYs future use in palliative care. The fact that the national and international meetings were conducted separately can be seen as both a strength and a weakness: national issues could be addressed in the national meeting (strength), yet national and international experts could not interact (weakness).

## Conclusions

This paper offers an international expert consensus on the earlier identified QALY issues [12], and advises on the QALY’s future use in palliative care. As opinions of international experts were gathered using a systematic approach, it is particularly well-informed. As long as no suitable alternative is available for the EQ-5D, other tools can serve as basis for the EQ-5D. Valuation of time should be incorporated in the QALY’s ‘Q’, so that the linear ‘clock time’ can stay in the ‘LY’ component. Even though the QALY currently does not suffice, a generic outcome measure of effectiveness is essential to justly allocate healthcare resources.

## Data Availability

The datasets used and/or analysed during the current study are available from the corresponding author on reasonable request.

## Abbreviations

CEA: Cost-effectiveness analysis
EQ-5D: EuroQol-5D
NGT: Nominal Group Technique
QALY: Quality-Adjusted Life Year
QoL: Quality of Life
